# Clear-Cell Adenocarcinoma of Vesical Origin: A Case Study of Metastatic Disease Treated with Chemotherapy

**DOI:** 10.1155/2010/426973

**Published:** 2010-10-17

**Authors:** Carolina Pena Álvarez, Isabel Lorenzo Lorenzo, Silvia Varela Ferreiro, Rosa Pardavila Gómez, Hae Jin Suh Oh, Andrea Saénz de Miera Rodríguez, Marta Covela Rúa, Wilver Federico Carbonell Luyo, Francisco Ramón García Arroyo, Pedro López Clemente, Patricia Palacios Ozores, Manuel Constenla Figueiras

**Affiliations:** ^1^Servicio de Oncoloxía Médica, Complexo Hospitalario de Pontevedra, Rúa Loureiro Crespo, 2, 36001 Pontevedra, Spain; ^2^Servicio de Oncoloxía Médica, Complexo Xeral Calde de Lugo, Rúa Doutor Ochoa s/n, 27004 Lugo, Spain; ^3^Servicio de Anatomía Patológica, Complexo Hospitalario de Pontevedra, Rúa Loureiro Crespo, 2, 36001 Pontevedra, Spain

## Abstract

Vesical clear cell adenocarcinoma is an uncommon tumour. The description of nearly all published cases focuses on histological issues, providing few clinical particulars and limited followup. The treatment choice is resection. No publications have been found regarding systemic treatments for advanced disease. We present a case of metastatic clear cell adenocarcinoma of the bladder treated with chemotherapy.

## 1. Introduction

Most of the published cases of metastatic clear-cell adenocarcinoma of the bladder describe women in their fifties or sixties who underwent total cystectomy as the only treatment [1]. This disease has also been described, to a lesser extent, in both men and in an age range between 19 and 80 years old. Most authors are typically specialists in pathology, and the publications focus on the peculiar histology of the disease and a study of the histogenesis as of two possible origins: mesonephric or Müllerian. The most common locations are the neck of the bladder, the urethra and the posterior wall, and there is often an urothelial component of the tumour [2]. Thus, it has been suggested that the tumours are derived from the mesonephros. The theory of Müllerian origin is based on three factors: its morphological similarity to tumours of this lineage within the female genital tract, on the fact that the tumors are largely found in women, and its association with endometriosis [3]. Immunohistochemical analysis of tumour samples supports both theories, so it has been assumed that the embryonic origin varies with each case [4].

From a histological perspective, it is also important to consider non invasive clear-cell adenoma as a differential diagnosis [5]. Both conditions are well characterised from a morphological viewpoint because they are recognisable without further study by light microscopy [6]. Nevertheless, both lesions present PAX8 expression that can help to differ them from urothelial carcinoma [7].

The clinical presentation is the same as that of urothelial tumours: mictional syndrome, acute urinary retention, and/or haematuria [1].

The usual treatment is surgical, with partial or radical cystectomy [1]. The subsequent published followup is short—rarely as long as 10 months—with the longest being 30 months. As such, the prognosis of this entity is difficult to estimate.

A single case of locally advanced tumour treated by pelvic exenteration did not recur during 30 months of followup [8]. In another case of extravesical spread, in which surgical treatment was not contemplated, the evolution was very poor, and the patient died eight months after diagnosis due to metastatic disease [9]. Only one case of radiotherapy treatment alone has been described, resulting in complete response of the localised disease [10].

Experience with chemotherapy is limited to an adjuvant setting. The drugs used include Cisplatin and 5-Fluoracil [1], Carboplatin and Methotrexate [11], and Adriamycin and Cyclophosphamide [12], with or without radiotherapy. However, because all of these instances are cases of adjuvant treatment and had little followup, we are unable to evaluate the disease sensitivity to chemotherapy.

## 2. Case Description

**Figure 1:**
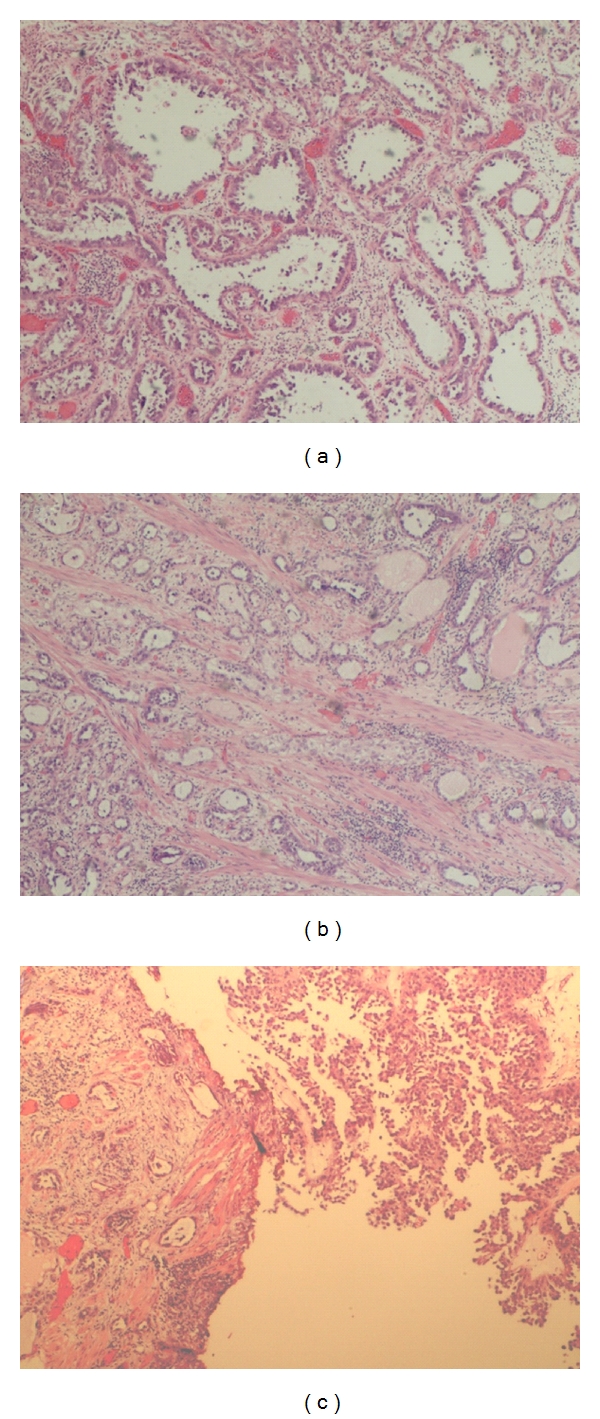
(a) Clear-cell adenocarcinoma (mesonephric). Glandular growth pattern with the presence of atypical hobnail cells. (b) Clear-cell adenocarcinoma (mesonephric). Invasion of the muscular layer of the bladder wall. (c) Sample from transurethral resection with transitional and clear-cell adenocarcinoma combination.

A 49-year-old woman developed mictional syndrome attributed to infection in September 2005. The symptoms did not improve with empiric antibiotic treatment, and she was referred to the Urology Department. She was diagnosed with a vesical tumour by cystoscopy in June 2006. It was resected by a transurethral resection, and a report described a mesonephric clear-cell adenocarcinoma (M9110/1 in the ICD-9-CM classification for malignant tumour morphology). The sample did not include the muscular layer of the bladder. Computerised tomography (CT) imaging was performed, revealing a thickening of the vesical wall and bilateral adnexal cystic structures. The transurethral resection was repeated, whereby the sample obtained was consistent with clear-cell adenocarcinoma (Figure 1a) with infiltration into the muscular layer (Figure 1b) of the bladder (pT2). It was associated with areas of high grade transitional cell carcinoma non-invasive (Figure 1c). The tumour was located within the neck of the bladder and the left half of the trigone. A CT scan demonstrated retroperitoneal nodes measuring less than one centimetre and iliac adenopathy measuring two centimetres. The patient underwent further surgery on November 9, 2006 with resection of an endometriosic ovarian cystic structure and an iliac adenopathy, which was identified during surgery as metastatic, and thus the surgery ended. The adenopathy was entirely infiltrated by clear-cell adenocarcinoma, without transitional component.

After recovery, the patient was referred to the Medical Oncology Department. During the first visit, she presented a PS (ECOG) of 1, with symptoms derived from the vesical condition. In view of the lack of bibliographical data concerning chemotherapy in metastatic disease, it was decided to use a regimen for urothelial carcinoma of the bladder with Cisplatin and Gemcitabine. Radiotherapy was not considered because nodes were present outside of the radiation field. During the fifth cycle, Cisplatin was replaced with Carboplatin due to a poorly controlled emetic syndrome. The best response after six cycles was radiological stabilisation, and the treatment was terminated. Radiological and clinical progression was observed three months later. Thus, a second line of treatment was initiated with Docetaxel, of which the patient received a total of five cycles, and with radiological stabilisation after the third and subsequent progression. Treatment was suspended in December 2007, at which time it was decided to administer symptomatic and support treatment.

In April 2008, the patient complained of back pain. A CT scan revealed progression of the tumour into the primary location and at nodal, liver, and bone level, with medullary compression at D12. She was urgently administered palliative radiotherapy and obtained one month of clinical improvement.

She subsequently presented with progressive decline in her overall status, including a gradual increase in pain, macroscopic haematuria, anaemia, and secondary obstructive clinical symptoms. She was radiated for palliative/haemostatic purposes in August 2008, with new clinical improvement. She subsequently died on November 6, 2008 as a result of tumour progression.

## 3. Conclusion

The combination of clear-cell and transitional adenocarcinoma in this case does not allow to assure the role of each histology in determining the outcome and the poor response to therapy. Although we can consider that the metastasic adenopathy was exclusively positive for clear-cell adenocarcinoma and that the transitional component was not infiltrative, as indications of the responsability of the clear-cell component in the poor outcome.

In the case of this patient, the disease was not sensitive to chemotherapy. Although more than 24 months of survival after diagnosis of metastatic disease is longer than the outcome typically described in the literature, it cannot be attributed to the chemotherapy treatment, because no objective response or clinical improvement was observed when the two lines of chemotherapy were administered. However, the patient improved on two occasions in response to the palliative radiotherapy treatment.

From a pathological perspective, a combination of mixed histology and endometriosis might be of interest because each of these support one of the two theories concerning the disease histogenesis. Unfortunately, the fact that the endometriosis was only in the ovary does not allow to connect it with the neoplastic disease, and this combination could be a sporadic association.
